# Reviewer acknowledgement 2012

**DOI:** 10.1186/1471-213X-13-5

**Published:** 2013-02-22

**Authors:** Philippa K Harris

**Affiliations:** 1 BioMed Central, 236 Gray's Inn Road, London, WC1X 8HB, UK

## Abstract

**Contributing reviewers:**

The editors of *BMC Developmental Biology* would like to thank all our reviewers who have contributed to the journal in Volume 12 (2012).

## 

Markus Affolter

Switzerland

Barbara Ahlemeye

Germany

Sara Ahlgren

USA

Takanori Amano

Japan

Raquel Andrade

Portugal

Francesco Argenton

Italy

Hiroshi Asahara

Japan

Katie Ayers

Australia

William Bates

Canada

Aaron Bell

USA

Thomas Boulin

France

Paola Bovolenta

Spain

Bruce Brandhorst

Canada

Thomas Braun

Germany

Melissa Brewster

USA

Jeremy Brockes

UK

Susan, Brown

USA

Robert Burke

Canada

Richard J Chaillet

USA

Wai Yee Chan

China

Chieh Chang

USA

Julia Chang

USA

Gavin Chapman

Australia

Kefei Chen

Australia

Jang-Soo Chun

South Korea

Joseph Corbo

USA

Stephen Crews

USA

Nicolas David

France

Roberta Davoli

Italy

Dirk G. de Rooij

Netherlands

Maria Donoghue

USA

Karel Dorey

UK

Richard Dorsky

USA

Wolfgang Drieve

Germany

Thomas Drysdale

Canada

Aude Dupré

France

Mototsugu Eiraku

Japan

Ralf Erdmann

Germany

Marta Fiorotto

USA

Ann Foley

USA

Alison Frand

USA

Nicolas Frankel

Argentina

Markus Friedrich

USA

Brigitte Galliot

Switzerland

José E. García-Arrarás

Puerto Rico

Richard Gomer

USA

Valerie Gouon-Evans

USA

Francois Graner

France

Andy Greenfield

UK

Alexander Gruhl

UK

Michael Hadfield

USA

Matthew Harris

USA

Shigeo Hayashi

Japan

Toshinori Hayashi

Japan

Robert Hill

UK

Bernadette Holdener

USA

Matthew Holley

USA

William Holmes

UK

Xun Huang

China

Isabelle Hue

France

Shigeho Ijiri

Japan

Tohru Iseto

Japan

Krzysztof Jagla

France

Hongbin Ji

China

Gabor Juhasz

Hungary

Koichi Kawakami

Japan

Kevin Kemp

UK

Abderrahman Khila

Canada

Kinarm Ko

South Korea

Marta Kostrouchova

Czech Republic

Ulf Krause

USA

Veit Krenn

Germany

Oscar Lee

Taiwan

Yonghe Li

USA

Robert Li

USA

Yi Li

USA

John Loughlin

UK

Mark Maconochie

UK

Moises Mallo

Portugal

Jocelyn McDonald

USA

Carolina Minguillon

Spain

Yuji Mishina

USA

Ravi Misra

USA

Nuno Monteiro

Portugal

Coleen Murphy

USA

Masahisa Nakamura

Japan

Alex Nechiporuk

USA

Phillip Newmark

USA

Wen Ning

China

Angelika A. Noegel

Germany

Joji Otaki

Japan

Michael Parsons

USA

Nipam Patel

USA

Achim Paululat

Germany

Catherine Pears

UK

Miguel Perez-Enciso

Spain

Peter Pfeffer

New Zealand

Jim Posakony

USA

Jo Anne Powell-Coffman

USA

Benjamin Prud'homme

France

Patricia Purcell

USA

Mary Reyland

USA

Gail Risbridger

Australia

Ann Rougvie

USA

Stéphane Roy

Canada

Florence Ruggiero

France

Susan Sadler

USA

Yumiko Saga

Japan

Charles Sagerstrom

USA

Patricia Salinas

UK

Leandro Sastre

Spain

Ken Sawai

Japan

Pauline Schaap

UK

Heide Schatten

USA

Fritz Schick

Germany

Reinhard Schuh

Germany

Richard Schultz

USA

David Sedmera

Czech Republic

Amanda Simcox

USA

Amit Singh

USA

Charles Singleton

USA

Uwe Straehle

Germany

Gustav Strijkers

Netherlands

Jennifer Stueckle

USA

Takayuki Suzuki

Japan

Nektarios Tavernarakis

Greece

Miguel Torres

Spain

Kim Tremblay

USA

Jun Udagawa

Japan

Susannah Varmuza

Canada

Srinivasan Vijayaraghavan

USA

Stephane Vincent

France

Irma Virant-Klun

Slovenia

Talila Volk

Israel

Stephen Voss

USA

Songlin Wang

China

Fen Wang

USA

Deneen Wellik

USA

Christopher West

USA

Rob White

UK

Dagmar Wilhelm

Australia

Carmen Williams

USA

Jeffrey Williams

UK

Li Xin

USA

Nan-Jie Xu

China

Xiaolei Xu

USA

Viravuth Yin

USA

Christos Zervas

Greece

Jue Zhang

USA

